# Analysis on Short-Term Outcomes for Cerebral Protection Treatment in Post Severe Traumatic Brain Injury Patients: A Single Neurosurgical Centre Study

**DOI:** 10.21315/mjms2024.31.2.12

**Published:** 2024-04-23

**Authors:** Ahmad Fikri Muhammad Mustafa, Laila Ab Mukmin, Mohd Zulfakar Mazlan, Abdul Rahman Izaini Ghani, Wan Mohd Nazaruddin Wan Hassan, Mohamad Hasyizan Hassan

**Affiliations:** 1 Department of Anaesthesiology and Intensive Care, School of Medical Sciences, Universiti Sains Malaysia, Kelantan, Malaysia; 2 Hospital USM, Universiti Sains Malaysia, Kelantan, Malaysia; 3 Department of Neurosciences, School of Medical Sciences, Universiti Sains Malaysia, Kelantan, Malaysia

**Keywords:** traumatic brain injury, cerebral protection, Glasgow Coma Scale, Glasgow Outcome Scale, neurocritical care

## Abstract

**Background:**

Severe traumatic brain injury (TBI) is a leading cause of disability worldwide and cerebral protection (CP) management might determine the outcome of the patient. CP in severe TBI is to protect the brain from further insults, optimise cerebral metabolism and prevent secondary brain injury. This study aimed to analyse the short-term Glasgow Outcome Scale (GOS) at the intensive care unit (ICU) discharge and a month after ICU discharge of patients post CP and factors associated with the favourable outcome.

**Methods:**

This is a prospective cohort study from January 2021 to January 2022. The short-term outcomes of patients were evaluated upon ICU discharge and 1 month after ICU discharge using GOS. Favourable outcome was defined as GOS 4 and 5. Generalised Estimation Equation (GEE) was adopted to conduct bivariate GEE and subsequently multivariate GEE to evaluate the factors associated with favourable outcome at ICU discharge and 1 month after discharge.

**Results:**

A total of 92 patients with severe TBI with GOS of 8 and below admitted to ICU received CP management. Proportion of death is 17% at ICU discharge and 0% after 1 month of ICU discharge. Proportion of favourable outcome is 26.1% at ICU discharge and 61.1% after 1 month of ICU discharge. Among factors evaluated, age (odds ratio [OR] = 0.96; 95% CI: 0.94, 0.99; *P* = 0.004), duration of CP (OR = 0.41; 95% CI: 0.20, 0.84; *P* = 0.014) and hyperosmolar therapy (OR = 0.41; CI 95%: 0.21, 0.83; *P* = 0.013) had significant association.

**Conclusion:**

CP in younger age, longer duration of CP and patient not receiving hyperosmolar therapy are associated with favourable outcomes. We recommend further clinical trial to assess long term outcome of CP.

## Introduction

Traumatic brain injury (TBI) is a leading cause of persistent disability worldwide. The likelihood of suﬀering disability increases with severity of TBI. In European countries, an overall incidence of 262 hospitalisations/100,000 inhabitants per year and 10% of the cases is severe TBI leading to variety of cognitive, emotional and neurological disabilities (1, 2). In Malaysia, TBI is the second most common cause of intensive care unit (ICU) admission and the 6th causes of hospital mortality (3).

A severe TBI ideally admitted to ICU/neurocritical unit for cerebral protection (CP). CP is a combination of patient management which includes intubation and ventilation to protect the airway and preventing hypoxia and hypercarbia, maintaining normal intravascular volume and electrolytes, optimising sedation and analgaesia, preventing fever and maintaining of ideal cerebral perfusion pressure to protect the brain from further insults and preventing secondary brain injury (4). This CP therapy are carried out based on guidelines produced by the Brain Trauma Foundation (5). However, the guidelines are too general with wide variety of selections and non-specific causing variation in performing CP worldwide. Thus, selection of medication and certain specific therapy are based on the centres and decision made by intensivist, neurosurgeon and neuro-anaesthesiologist who manage the patient.

Patient’s neurological status after TBI is greatly dependent on numerous factors, including severity of injury (mild, moderate or severe), mechanism (blunt or penetrating injury) and other patient factors (6). Glasgow Outcome Scale (GOS) is the mostly used to measure outcome after TBI. This tool measures functional ability of patient, which scored from 1 to 5. GOS 1–3 is categorised to unfavourable outcome whereas GOS 4 and 5 is favourable outcome. Favourable outcome is defined as ability of the patient to be neurologically independent after the TBI. Wilson et al. (7) concluded that assessment of the GOS using standard format with a written protocol is practical and reliable. The weighted average mortality for severe TBI was 39% and for an unfavourable outcome on the GOS was 60% according to a 2012 meta-analysis (8).

There are variations of practice of CP by local physicians, thus no complete data available to link CP with the outcomes so far. This study justified use of proper technique of CP to reach certain target, i.e. deep sedation with or without bispectral index (BIS) monitoring, optimisation of blood pressure with aimed CPP > 55, achievement of normal blood gas and electrolyte parameters to prevent secondary brain insult. Although CP does not totally prevent unfavourable GOS outcome, there was no local data reporting the outcome of CP and the factors associating with favourable outcome. Hence, this study aimed to evaluate the outcomes of severe TBI after CP which initially categorised into death and alive, then favourable and unfavourable outcomes and finally factors associated with favourable outcomes after CP at ICU discharge and 1 month after discharge. This study aimed to identify mortality rate and GOS of the TBI patients post CP at ICU discharge and 1 month after discharge and determine factors acssociated with favourable outcome.

## Methods

### Study Designs

This study included 92 patients aged 18 years old–65 years old who diagnosed with severe TBI based on Glasgow Coma Scale (GCS) 8 and below which then admitted to ICU for CP therapy. Exclusion criteria were patients with pre-existing neurological medical problems that could affect the outcome such as cerebral vascular accident, seizures, tumours or malignancy and neurological disability. Patients with other major injury involving intra-abdominal, intrathoracic and spinal cord were also excluded as it may affect the outcomes. Since all patients were not able to give their own consent due to their limiting GCS, the written informed consent was obtained from patient’s relative for those who included in the study.

All patients who were diagnosed with severe TBI admitted to ICU and received CP therapy were recruited based on purposive sampling method. CP treatment was given based on the Brain Trauma Foundation guidelines (5). If the patient underwent surgery, the CP management was started after the operation. If the patient did not have any surgery done, the initiation time of CP management was recorded; whether less or more than 1 h after injury. As for CP management, for sedation protocol, patients were given infusion of intravenous (IV) fentanyl (0.5 mcg/kg/h–1 mcg/kg/h) and IV propofol (0.5 mg/kg/h–4 mg/kg/h) to maintain Ramsay Agitation and Sedation Score (RASS) of −4 to −5 and total duration of CP was determined by neurosurgeon in charged. Haemodynamically, the mean arterial pressure (MAP) was kept at targeted value of 80 mmHg. Infusion of IV noradrenaline started and titrated accordingly to achieve this target. Hourly pupillary charting was done. The continuous intercranial pressure (ICP) monitoring was also performed in patient with extraventricular drain (EVD). The EVD is connected via catheter and transducer system, Medtronic Exacta^TM^ drainage system and continuous pressure recorded with ICP monitor, Philips Intellivue MX800. Other components of CP include hyperosmolar therapy, barbiturate coma and hypothermic therapy were given based on assessment and judgement by the treating neurosurgeon. The reassessment computed tomography (CT) imaging was performed after completion of the CP.

**Figure 1:**
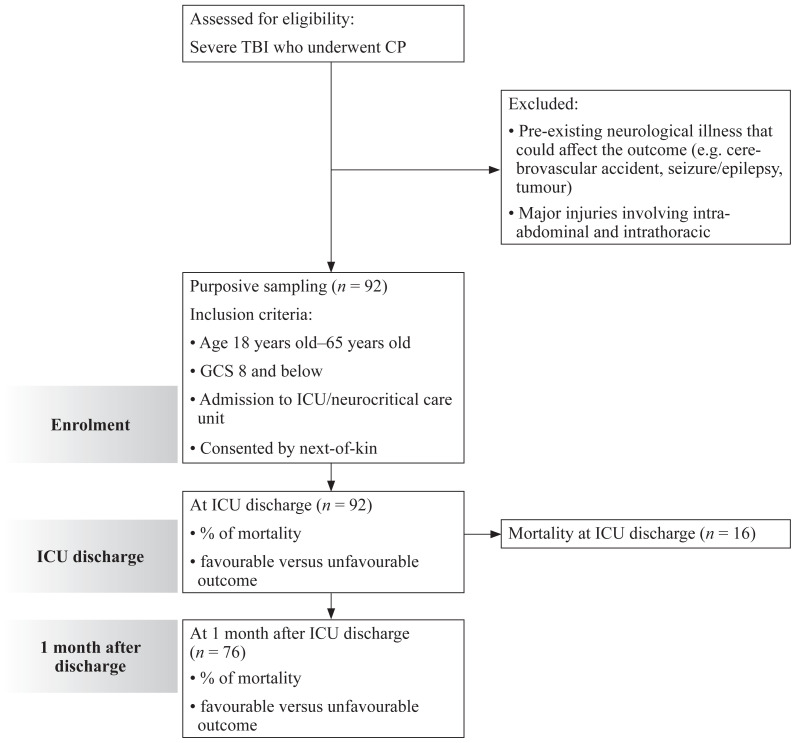
STROBE diagram

Patients were assessed upon ICU discharge using GOS (7), whereby score 1 = death, 2 = vegetative state, 3 = severe disability, 4 = moderate disability and 5 = good recovery. Patients were assessed again using this scale 1 month after ICU discharge by phone call or during follow-up clinic. Factors associated with the outcomes were evaluated from these findings. The STROBE diagram for observational study as shown in Figure 1.

### Measurement of Outcomes

The outcomes of this study were based on GOS which consist of scores 1–5. Score 1 for death, score 2 for vegetative state which defined as condition which patient unable to interact with environment or surrounding. Score 3 for severe disability, defined as patient able to follow commands but unable to live independently. Score 4 for moderate disability which patient are able to live independently but unable to return to work or school and score 5 for good recovery, defined as patient able to return to work or school. This score then categorised into favourable outcome for scores 4–5 and unfavourable outcome for scores 1–3. The short-term outcome for the study is defined as outcome at ICU discharge and 1 month after ICU discharge. All patients were followed-up at 1 month after ICU discharge, and reassessment of GOS at 1 month after discharged was done via phone interview with the patients’ caretakers or during neurosurgical clinic follow-up appointment. There was not further follow-up after 1 month assessment.

Several factors associating with the outcome were identified. The factors variables includes: age of the patient, presenting GCS of the patient (pre-resuscitated and pre-intubated GCS), patient’s gender, total time interval before initiation of CP management (less than 1 h after injury versus more than 1 h after injury if the patient was not operated versus after surgery), total duration of CP management (24 h versus 48 h), presence versus absence of surgery (craniotomy/craniectomy and/or extraventricular drainage (EVD), presence versus absence of hyperosmolar therapy using hypertonic saline 7.4% or mannitol 20% during CP management and presence versus absence of mild hypothermia therapy to 34 °C–35 °C during CP management.

### Sample Size

The sample size was estimated using the G-power 3.1 software. *F*-test, linear multiple regressions: fixed model, *R*^2^ deviation from zero was selected. Given α = 0.05, power = 0.8 and effect size = 0.15. Number of predictors to be evaluate in this study is 5, thus the total sample size required for this study is 92.

### Statistical Analysis

Data analysis in this study was conducted using STATA/SE 12.0 (StataCorp, College Station, TX, USA). Continuous variables were presented in mean (standard deviation [SD]) or median (interquartile range [IQR]) depending on the normality of data distribution. Categorical data was presented as frequency and percentage.

In evaluating the factors associated with favourable outcome upon ICU discharged and at 1 month after, Generalised Estimation Equation (GEE) based marginal models was adopted. Prior to analysis, data was first transformed into long format. Because the response outcome was binary with two possible outcomes (favourable versus unfavourable), the binomial distribution and logit link function were specified. The first step in GEE modelling strategies was to conduct bivariate GEE. Variables that were found to be statistically significant (*P* < 0.05) and additional variables that might plausibly affect the dependent variable were considered as candidate variables in multivariate GEE. Subsequently, parameters of the correlation structure were estimated, giving approximate covariance matrix of the parameter estimates and observations. Calculation based on Quasi-likelihood under the independence model criterion (QIC) was used to select the best working correlation structure in GEE analyses. Result showed that the unstructured correlation structure has the smallest QIC and thus was chosen as the preferred correlation matrix. Then, goodness of fit (GOF) test and residual analysis was carried out to assess the fit of the model. For GOF test, chi-squared GOF test was adopted. A statistically not significant *P*-value obtained (*P* = 0.3871) concluded that the model was fit. The final model was obtained and result were interpreted in terms of odds ratio (OR). All probability values were two-sided. A *P*-value of less than 0.05 was considered statistically significant.

## Results

A total of 92 patients were included in this study. Only 76 patients were followed-up at 1 month after ICU discharge since 16 patients died during hospitalisation. The median age of patients suffering severe TBI in our study was 26 years old (IQR = 19–44.5 years old); 32.6% of the patients has underlying comorbidities and 68.5% of them underwent surgical procedures before ICU admission (Table 1).

Initiation of CP therapy was started mostly after surgical intervention in 62 patients (67.4%) but 30 patients (32.6%) were started on CP after 1 h of trauma and no patient received CP within 1 h after trauma (Table 2). For duration of CP therapy, 54 patients (58.7%) received therapy for 24 h while others 38 (41.3%) patients completed therapy for 48 h (Table 2). As for component in CP management, only 26 (28.3%) patients received hyperosmolar therapy and 4 (4.3%) patients received hypothermia therapy.

At ICU discharge, 16 patients (17.4%) were dead while 76 patients (82.6%) survived (Table 3). At ICU discharge, 68 patients (63.9%) were unfavourable in outcome while other 24 patients (26.1%) were favourable in outcome (Table 3). Seventy-six patients who survived at ICU discharge were followed-up after 1 month. All patients remained alive at 1 month post ICU discharge and 51 patients (67.1%) fall into favourable outcome while remaining 25 patients (32.9%) fall into unfavourable outcome (Table 3).

**Figure 2:**
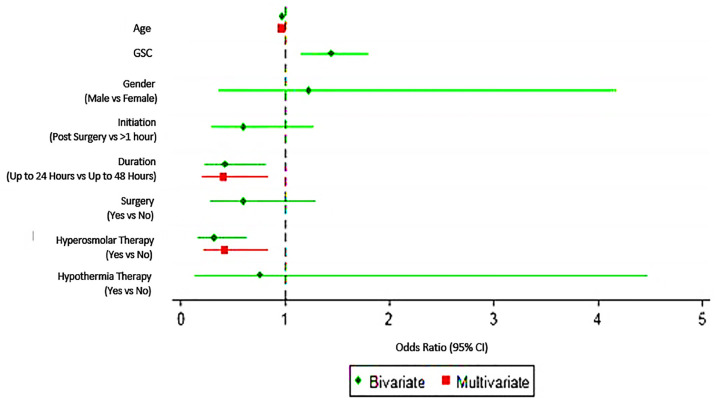
Factors associated with the outcome of CP treatment

All possible associating factors towards the outcome were evaluated i.e. initial GCS upon presentation, time to start CP, duration of CP, surgical intervention and components of CP. Out of these, age, duration of CP and hyperosmolar therapy have significant association with favourable outcome. For age, the OR indicates that for every one-year increase in age, the likelihood to have favourable outcome at ICU discharged and 1 month after ICU discharged reduces by approximately 0.96 times (OR = 0.96; 95% CI: 0.94, 0.99) (Table 4, Figure 2).

For duration, the OR indicates that as compared to 48 h CP, the likelihood to have favourable outcome at ICU discharged and 1 month after ICU discharged after CP for 24 h reduces by approximately 0.41 times (OR = 0.41; 95% CI: 0.20,0.84) (Table 4, Figure 2). For hyperosmolar therapy, the OR indicates that as compared to patient without any hyperosmolar therapy, the likelihood to have favourable outcome at ICU discharged and 1 month after ICU discharged reduces by approximately 0.41 times (OR = 0.41; 95% CI: 0.21, 0.83) (Table 4, Figure 2). Upon multivariate analysis, there was no significant association between GCS at presentation with the favourable outcome.

## Discussion

Following primary insult in severe TBI, secondary brain injury may occur as a result of increased intracranial pressure (ICP), intracranial bleeding, ischaemia, hypercarbia and hypoxia (9). This type of injury may be prevented or minimised by CP. Brain Trauma Foundations had produced a guideline for managing these kinds of patients which includes medical therapy, surgical interventions and monitoring (4).

In our study, median age of severe TBI patients were 26 years old. Similarly, local systematic review done found that average age of population is in 30s in 21 studies while 20s in another 15 studies (10). Male patients were greater in numbers and supported by meta-analysis done by Frost et al. (11) on prevalence of TBI in developing countries. The patients age and sex in this study were consistent with previous studies conducted locally and internationally. Ideally CP therapy should be initiated as soon after trauma. However, transportation of patient from site of injury to the hospital with tertiary facilities may delayed especially in developing countries. Patient may initially transport to nearby district hospital for early resuscitation and stabilisation before referral to these facilities. Decision for initiation of CP or surgical intervention prior to CP were made by neurosurgeon in the tertiary hospital. Duration of treatment also depends on decision by the managing team based on their assessment of patient and radiological findings. Usually duration of 24 h–48 h was given to the patient as it is a crucial time of development of secondary brain injury. To date, there is no specific study done in evaluating optimal duration of CP given to patients.

In this study, all patients received CPs. Some patients had received certain additional components of CP such as hyperosmolar therapy, thiopentone coma, hypothermic therapy and surgical interventions. As a result of these holistic approach, this study showed fatality rate of 17.4% at ICU discharge which is slightly less than mortality of 18% in systematic review in Europe (12). Higher fatality was seen in this systemic review because they compile all mortality over longer time frame which differ from our study that had shorter time limit to 1 month after ICU discharge. In our study, proportion of unfavourable outcome was 63.9% at ICU discharge. This higher proportion was mainly contributed by early ICU discharge from high turnover neurocritical care unit. The patients who were able to be liberated from ventilatory support, haemodynamically stable and fulfilled minimum ICU discharge criteria were transferred out to general ward and district hospital although the patients’ functional status were not fully recovered (13). However, proportions of favourable outcome improved to 67.1% 1 month after ICU discharge while unfavourable outcome dropped to 32.9% over same time point (Table 3, Figure 1). These findings correspond to study done by Aarabi et al. (14) and Timofeev et al. (15).

Several factors had been evaluated for associations with this favourable outcome. Factors such as GCS on arrival, time of CP initiation, duration of CP, surgical interventions and component of CP which are hyperosmolar therapy, hypothermia and thiopentone coma were evaluated. Age had a strong association which showed for every 1 year increase in age, it is less likely to experience favourable outcome at ICU discharged and 1 month after ICU discharged. Age is non-modifying demographic factor which has a strong association with outcomes in severe TBI patients post-treatment. Older age always associated with unfavourable outcome (16). This poor outcome most probably due to physiological changes in central nervous system (CNS) that occur as age increasing (17, 18). These changes may lead to reduction in functionality, cognitive, memory and slower or irreversible recovery after certain insult or even after exposure to medication with CNS effect such as general anaesthesia. In additions, elderly population may also have metabolic and poorer cardiac reserve and functions delaying process of recovery (18).

Hyperosmolar therapy is one of the component in CP which were used to reduce ICP and in patient with brain herniation syndrome. In our study, we found out that patient receiving hyperosmolar therapy had less likely to experienced favourable outcome compare to patient without hyperosmolar therapy. In contrast, a study done by Roquilly et al. (19) found that there were no statistically significant in better outcomes of TBI patients receiving continuous hypertonic saline compare to patient in standard care group. Because of this lack of evidence of this therapy on the clinical outcome, there is no specific recommendation for this therapy in current 4th edition Guidelines for Management of Severe TBI (5). In terms of type of hyperosmolar therapy which were hypertonic saline and mannitol had no significant association with the favourable outcome as similar to the study done by Cottenceau et al. (20).

Based on the multivariate analysis, the baseline GCS was not associated with changes in outcome. The patients with longer duration of CP were more likely to experience favourable outcome at ICU discharge and 1 month after ICU discharge. Thus, this finding supports longer duration of CP in patients with severe TBI to improve the GOS at ICU and hospital discharge. Traditionally, the CP was only performed for 24 h and usually stopped after repeated CT brain at 24 h post-injury. Some patients’ CP were prolonged based on clinician recommendation. Our study found significant association between longer duration of CP with improved neurological outcome. Thus, we suggest prolonged duration more than 24 h of CP especially in patients with severe TBI. Serial CT scan might also be needed before decision to terminate the CP is made. Further randomised controlled trial would be needed to support our finding.

## Conclusion

CP therapy gives a favourable outcome in severe TBI especially seen 1 month after ICU discharge. Younger age, longer duration of CP and patients who did not received hyperosmolar therapy was associated with statistically significant association with favourable outcome in severe TBI patients.

### Limitation and Suggestion

Our study was limited with patients underwent CP in a single tertiary centre. Apart from that, some patients were also referred from other peripheral hospital that might not institute adequate measures for CP which subsequently influence the outcome of the study. There was also heterogenicity in the patients since not all of them had ICP guided CP especially those who did not underwent surgery. Moreover, we only evaluate the short-term outcome for 1 month post ICU discharge. We suggest a multicentre interventional study involving patient who optimally undergoing CP with longer evaluation time for future studies.

## Figures and Tables

**Table 1:** Demographic profile of severe TBI patients underwent CP

Parameter	Total patient (*n* = 92)	%
Age (years old)	26 (19–44.5)^a^	
Gender		
Female	10	10.9
Male	82	89.1
Weight (kg)	67.9 (12)^b^	
Height (cm)	170 (165–170)^a^	
Types of comorbidity		
Hypertension	11	12
Diabetes mellitus	7	7.6
Dyslipidaemia	3	3.3
Chronic kidney disease	1	1.1
Others	8	8.7
GCS at presentation		
3	17	18.5
4	5	5.4
5	7	7.6
6	6	6.5
7	15	16.3
8	42	45.7
CT brain findings		
EDH	14	15.2
SDH	21	22.8
SAH	13	14.1
Combination	44	47.8
Surgical procedure		
Yes	63	68.5
Type of surgery		
EVD	17	27

Craniotomy+Evacuation of clot+EVD	15	23.8
Craniectomy+Evacuation of clot+ EVD	31	49.2

Notes:

a Data in median (IQR);

b Data in mean (SD);

GCS = Glasgow Coma Scale; CT = computed tomography; EVD = extraventricular drain

**Table 2:** Initiation, duration and components of CP management

CP management	Total patient (*n* = 92)	%
Initiation of CP		
Post-surgical procedure	62	67.4
More than 1 h	30	32.6
Duration of CP		
completed 24 h	54	58.7
completed 48 h	38	41.3
Hyperosmolar therapy		
Mannitol 20%	19	20.7
Hypertonic saline 7.4%	7	7.6
Hypothermia therapy	4	4.3

**Table 3:** Mortality and GOS of severe TBI after CP at ICU discharge and at 1 month after ICU discharge

	Total patient	%
At ICU discharge (*n* = 92)		
Mortality	16	17.4
GOS 1–3	68	63.9
GOS 4 and 5	24	26.1
At 1 month (*n* = 76)		
Mortality	0	0
GOS 1–3	25	32.9
GOS 4 and 5	51	67.1

**Table 4:** Factors associated with favourable outcome at ICU discharge and 1 month after ICU discharge

Factors	Bivariate, OR (95% CI)	*P*-value	Multivariate, OR (95% CI)	*P*-value
Age (years old)	0.969 (0.945, 0.994)	0.015	0.961 (0.936, 0.988)	0.004
GCS	1.437 (1.145, 1.803)	0.001		
Gender				
Male	1			
Female	1.222 (0.357, 4.181)	0.750		
Initiation of CP				
Less than 1 h	1			
More than 1 h	0.597 (0.281, 1.271)	0.181		
Duration of CP				
24 h	0.420 (0.217, 0.812)	0.010	0.410 (0.200, 0.838)	0.014
48 h	1			
Surgical intervention				
Yes	0.599 (0.277, 1.296)	0.193		
No	1			
Hyperosmolar therapy				
Yes	0.315 (0.157, 0.632)	0.001	0.4148 (0.206, 0.832)	0.013
No	1			
Hypothermia therapy				
Yes	0.756 (0.128, 4.476)	0.758		
No	1			
